# The Earliest Case of Extreme Sexual Display with Exaggerated Male Organs by Two Middle Jurassic Mecopterans

**DOI:** 10.1371/journal.pone.0071378

**Published:** 2013-08-14

**Authors:** Qi Wang, Chungkun Shih, Dong Ren

**Affiliations:** Key Lab of Insect Evolution and Environmental Change, College of Life Sciences, Capital Normal University, Beijing, China; University of Utah, United States of America

## Abstract

**Background:**

Many extant male animals exhibit exaggerated body parts for display, defense or offence in sexual selection, such as male birds of paradise showing off colorful and elegant feathers and male moose and reindeers bearing large structured antlers. For insects, male rhinoceros and stag beetles have huge horn-like structure for fighting and competition and some male *Leptopanorpa* scorpionflies have very long abdominal terminal segments for sexual display and competition. Fossil records of insects having exaggerated body parts for sexual display are fairly rare. One example is two male holcorpids with elongate abdominal segments from sixth (A6) to eighth (A8) and enlarged male genitalia from Eocene, suggesting evolution of these characters occurred fairly late.

**Principal Findings:**

We document two mecopterans with exaggerated male body parts from the late Middle Jurassic Jiulongshan Formation in northeastern China. Both have extremely extended abdominal segments from A6 to A8 and enlarged genitalia, which might have been used for sexual display and, to less extent, for fighting with other males in the competition for mates. Although ***Fortiholcorpa paradoxa*** **gen. et sp. nov.** and ***Miriholcorpa forcipata*** **gen. et sp. nov.** seem to have affinities with Holcorpidae, we deem both as Family Incertae sedis mainly due to significant differences in branching pattern of Media (M) veins and relative length of A8 for *F. paradoxa*, and indiscernible preservation of 5-branched M veins in hind wing for *M. forcipata*.

**Conclusions/Significance:**

These two new taxa have extended the records of exaggerated male body parts of mecopterans for sexual display and/or selection from the Early Eocene to the late Middle Jurassic. The similar character present in some *Leptopanorpa* of Panorpidae suggests that the sexual display and/or sexual selection due to extremely elongated male abdominal and sexual organs outweigh the negative impact of bulky body and poor mobility in the evolutionary process.

## Introduction

Holcorpidae is an enigmatic and controversial extinct family in Mecoptera, which has been documented and debated since 1878. Scudder placed *Holcorpa* in the family Panorpidae in 1878, because he considered that the scorpionfly was closely related to *Panorpa* Linnaeus, 1758 [1]. Since then, the discovery of more fossil specimens with 5-branched M (Media) veins in both the fore- and hind wings (vs. four-branched M veins in all Panorpidae) indicates the genus *Holcorpa* Scudder, 1878 does not belong to Panorpidae. Carpenter [2] considered it has closest affinities with Old World genera, as its body structure is similar to *Neopanorpa* and *Leptopanorpa* (Panorpidae), both of which are restricted to the Old World. Penny [3] and Martynova [4] suggested it should be placed into a separate family. Over years, many authors have subscribed to the view of not considering *Holcorpa* as a panorpid, and have mentioned the name “Holcorpidae” in some cases. It is Willmann [5] who provided a description distinguishing and defining the family as required by article 13 of the International Code of Zoological Nomenclature [6] for family names published after 1930. In 2010, Archibald distinguished *Holcorpa* from all known Panorpidae by a combination of diagnostic characters: (1) fore-, and hind wing media veins with five branches; (2) abdominal segments 6 (A6) to 8 (A8) elongate (both male, female), with A8 distinctly longest; (3) male: extended, slender dististyli, lacking basal tooth [7].

Fossil records of the Holcorpidae are fairly rare. Up to date, only one fossil genus *Holcorpa* with two species *Holcorpa maculosa* Scudder, 1878 and *Holcorpa dillhoffi* Archibald, 2010 within this family have been described. The first holotype specimen, *H. maculosa*, was described by Scudder [1]. The body is poorly preserved, incomplete beyond the eighth abdominal segment. The second specimen (allotype) was collected in 1907, which was identified as a male by Carpenter. The abdomen of the allotype is well preserved: the male genitalia clearly visible, with distinctive enlarged genital bulb and extended pincer-like gonostyli. Both fossils are from the Late Eocene of Florissant, Colorado, USA. The other species is *H. dillhoffi* from the Ypresian (Early Eocene) McAbee beds of British Columbia, Canada [7].

Compared to the holotype, the larger size of the allotype was explained by Carpenter [2] as sexual dimorphism by concluding that the holotype is a female, contrary to Scudder’s opinion [1], [7]. Based on new data and information from new fossil material of [7] and this study, we believe that the holotype should be considered as sex indeterminate due to lack of preserved terminalia. The sudden narrowing of A8, comparing with A7, is consistent with male specimens in [7] and this study.

Two new mecopterans, *Miriholcorpa forcipata* gen. et sp. nov. and *Fortiholcorpa paradoxa* gen. et sp. nov., are described herein. These specimens were collected from the Jiulongshan Formation at Daohugou Village of Ningcheng County in Inner Mongolia, China [8]–[11]. Based on M with five branches in both fore- and hind wings; sixth to eighth abdominal segments exceedingly elongate and genitalia enlarged, *F. paradoxa* gen. et sp. nov. seems to have affinities with Holcorpidae. But, we deem *F. paradoxa* gen. et sp. nov. as Family Incertae sedis mainly due to significant differences in branching pattern of Media (M) veins in hind wings; length of A8 slightly longer than that of A7 (vs. A8 distinctively longest); and lacking spurs at the terminal part of A6 (vs. two spurs present). We also deem *M. forcipata* gen. et sp. nov. as Family Incertae sedis, due to uncertainty whether its hind wing has 5-branched M veins.

The age of the Daohugou fossil-bearing beds is considered to be the late Middle Jurassic [12]–[14], ca. 165 Ma, corresponding to the Callovian–Bathonian boundary using a standard international time scale [15]. Daohugou fossil-bearing beds contain abundant exquisite Mecoptera fossils. Until now, many specimens of Pseudopolycentropodidae [16]–[18], Mesopsychidae [16], [19], Eomeropidae [20], Bittacidae [21], Aneuretopsychidae [16], [22], [23] and Cimbrophlebiidae [21], [24] have been reported from this locality.

## Materials and Methods

### Material

The fossil specimens were collected from the Jiulongshan Formation at Daohugou Village of Ningcheng County in Inner Mongolia, China. All specimens are deposited in the Key Lab of Insect Evolution & Environmental Changes, College of Life Sciences, Capital Normal University, Beijing, China (CNU, Dong Ren, Curator). No specific permits were required for the described field studies.

### Methods

**Figure 1:**
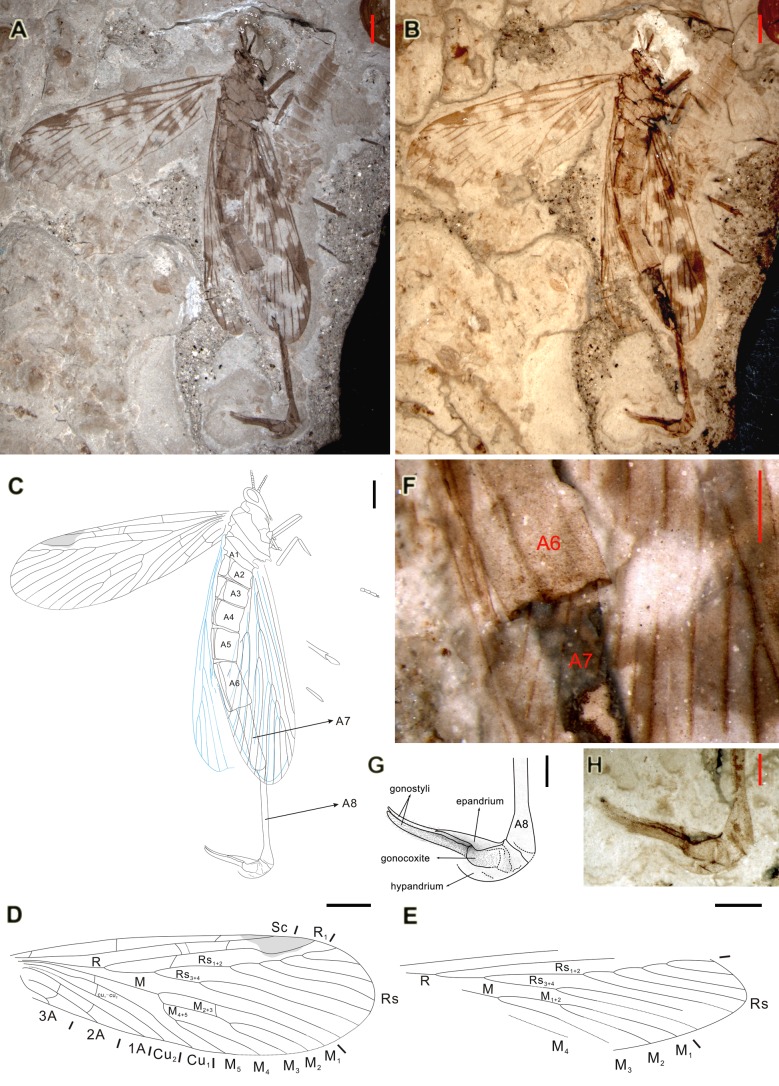
*Miriholcorpa forcipata* gen. et sp. nov., holotype CNU-MEC-NN-2012001. A, photograph of holotype; B, photograph of holotype under alcohol; C, line drawing of holotype; D, line drawing of the left forewing (flipped to right for easy comparison); E, line drawing of the hind wing, composite of both left and right wings; F, photograph of distal part of A6 showing absence of spurs, under alcohol; G, line drawing of genitalia; H, photograph of genitalia, under alcohol. Scale bars represent 2 mm in A–C, D and E, 1 mm in F, G and H.

**Figure 2:**
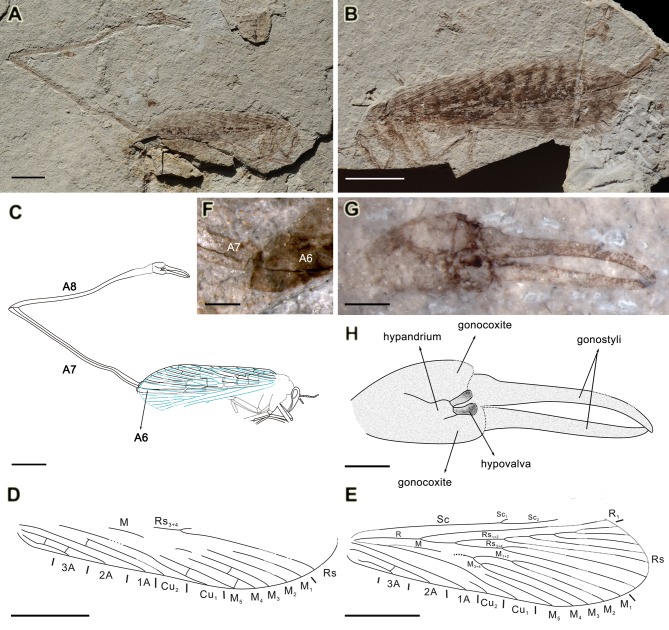
*Fortiholcorpa paradoxa* gen. et sp. nov., holotype CNU-NEU-NN-2012002P/C. A, photograph of part; B, photograph of counterpart; C, line drawing of part; D, line drawing of forewing of part; E, line drawing of hind wing of part; F, photograph of distal part of A6 showing absence of spurs and basal part of A7 showing the fusion line of tergite and sternite of A7, under alcohol; G, photograph of genitalia of part; H, line drawing of genitalia of part. Scale bars represent 5 mm in A–E, 1 mm in F, G and H.

**Figure 3:**
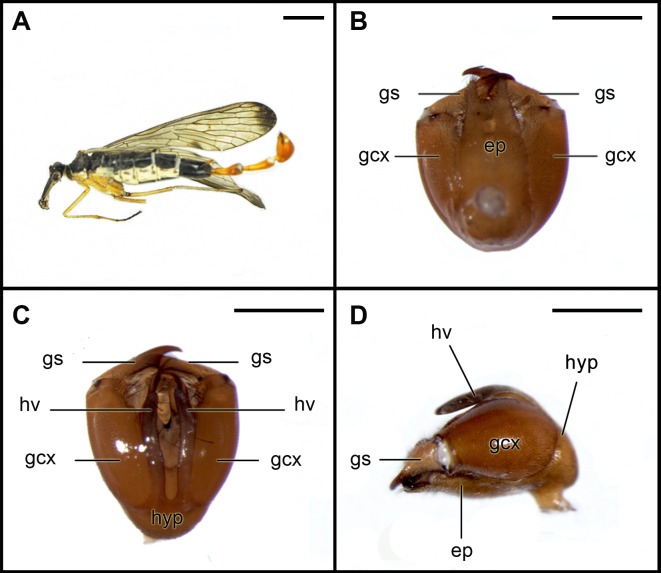
Photographs of a male specimen of *Panorpa dubia* Chou & Wang, 1981. Collected in Baotianman Nature Reserve of Nanyang City in Henan Province by Junhui Liang, Xiaoguang Yang, Huifeng Zhao, Chaofan Shi and Yan Zhu on June 10, 2011. A, the habitus; B–D. male genital bulb and gonostyli. B, dorsal view; C, ventral view; D, lateral view. ep, epandrium; gcx, gonocoxite; gs, gonostylus; hv, hypovalva; hyp, hypandrium. Scale bars represent 3 mm in A, 1 mm in B–D.

The specimens were examined using a Leica MZ12.5 dissecting microscope and illustrated with the aid of a drawing tube attachment or photographs. Photographs of Figs. 1A, B, F, H, Figs. 2F, G and Figs. 3B–D were taken using a Nikon SMZ1000 stereomicroscope. Photographs of Figs. 2A, B were taken using a Nikon D100 digital camera with a Nikkor 105 mm macro lens. Photograph of Fig. 3A was taken using a Nikon D500 digital camera with a Nikkor 105 mm macro lens. The line drawings were drawn by CorelDraw 12.0 and Adobe Photoshop CS5. We use the venational nomenclature of Willmann [5]. For Mecoptera systematics, we follow Willmann’s [5], [25] and Willmann & Novokshonov’s [26], except for Orthophlebiiidae and the genus *Orthophlebia*, which were subsequently revised by Hong & Zhang [27], [28].

### Nomenclatural Acts

The electronic edition of this article conforms to the requirements of the amended International Code of Zoological Nomenclature, and hence the new names contained herein are available under the Code from the electronic edition of this article. This published work and the nomenclatural acts it contains have been registered in Zoobank, the online registration system for the ICZN. The Zoobank LSIDs (Life Science Identifiers) can be resolved and the associated information viewed through any standard web browser by appending the LSID to the prefix “Http://zoobank.org”. The ISID for this publication is: urn:lsid:zoobank.org:pub:B2F83E59-492E-4B6C-81B8-59D501B4C301. The electronic edition of this work was published in a journal with an ISSN, and has been archived and is available from the following digital repositories: PubMed Central and LOCKSS.

## Results

### Systematic Palaeontology

Mecoptera Packard, 1886

Family Incertae sedis.

*Miriholcorpa* Wang, Shih & Ren gen. nov.

urn:lsid:zoobank.org:act:79F6D4BF-0D0C-4E02-88EE-660F7E5651D5

#### Type species

*Miriholcorpa forcipata* Wang, Shih & Ren sp. nov.

#### Etymology

The generic name is a compound word derived from a Latin word *Miri*-, meaning amazing, referring to the fact that the specimen is amazingly and beautifully preserved and *Holcorpa*, the type genus of Holcorpidae.

#### Diagnosis

In forewing, Sc reaching anterior margin almost at middle of pterostigma. Rs furcation at same level as M. Stem of Rs_3+4_ short. Stem of Rs_1+2_ 1.5 to 2.0 times as long as stem of Rs_3+4_. M_1_ simple. M_2+3_ forking to M_2_ and M_3_, M_4+5_ forking to M_4_ and M_5_. Only one crossvein between Cu_1_ and Cu_2_ (cu_1_-cu_2_).

#### Remarks

This genus has many diagnostic characters of Holcorpidae such as forewing R_1_ curving around pterostigma, meeting C on anterior wing margin well before apex, forewing M veins with five branches, abdominal segments 6 (A6) to 8 (A8) elongate with A8 distinctly longest; and male genitalia with gonostyli extended and slender. However, the new genus is different from the type genus *Holcorpa* of Holcorpidae in branching pattern of forewing M veins and spurs absent at the end of A6. In addition, it is not discernible whether hind wing has 5-branched M veins, or whether dististyli basal tooth is lacking [5], [7]. In Table 1, the venational characters of fore- and hind wings of this new genus are compared with two species of *Holcorpa* and three representative male species of genus *Orthophlebia* in Orthophlebiidae with elongate A6 to A8. This new genus is significantly different from the three representative orthophlebiids. Therefore, due to uncertainty whether hind wing of *Miriholcorpa forcipata* gen. et sp. nov. has 5-branched M veins, we deem this new genus as Family Incertae sedis, pending future discovery of new fossil specimens with clear hind wing venation. Note that Ohm stated the difference of branching numbers for forewing Rs_1+2_ may be a variation within a species [29].

**Table 1:** Comparison of venational characters of fore- and hind wings for species with lengthened male abdominal segments A6 to A8.

	Material ID number	Forewing, Stem of Rs_1+2_/stem of Rs_3+4_	Forewing, Rs forking vs. M forking	Forewing, Rs_1+2_	Forewing, M	Hind wing, Stem of Rs_1+2_/stem of Rs_3+4_	Hind wing, Rs forking vs. M forking	Hind wing, Rs_1+2_	Hind wing, M
***Holcorpa*** **Scudder, 1878, Holcorpidae Willmann, 1989**									
***H. maculosa*** ** Scudder, 1878**	AMNH 18887 (part) and UCM4494 (counterpart)	ca. 0.5 *	Significantly basal	4 pectinate	M_1+2_, M_3_, M_4+5_	ca. 0.5	Significantly basal	4 pectinate	M_1+2_, M_3_, M_4+5_
***H. dillhoffi*** ** Archibald, 2010**	RBCM-EH-2008-018-0001 (part only)	ca. 0.5	Significantly basal	4 pectinate	M_1_ furcation not preserved, M_3_, M_4+5_	ca. 0.5	Significantly basal	4 pectinate	
***Fortiholcorpa*** ** gen. nov., Family Incertae sedis**									
***F. paradoxa*** ** sp. nov.**	CNN-MEC-NN-2012002 P/C	N. A. **	N. A.		5 branches with unclear furcations	1	Slightly basal	5 pectinate	M_1+2_, M_3+4_, M_5_
***Miriholcorpa*** ** gen. nov., Family Incertae sedis**									
***M. forcipata*** ** sp. nov.**	CNN-MEC-NN-2012001 (part only)	1.65	Same level	5 pectinate	M_1_, M_2+3_, M_4+5_	1.36	Slightly basal	5 pectinate	M_1+2_, M_3,_ M_4_ M_5_ N. A.
***Orthophlebia*** ** Westwood, 1845, Orthophlebiidae Handlirsch, 1906**									
***O. nervulosa*** ** Qiao, Shih et Ren, 2012**	CNU-MEC-NN-2009617 (part only)	1	Significantly basal	6 pectinate	M_1+2_, M_3_, M_4+5_	0.5	Significantly basal	6 pectinate	M_1+2_, M_3+4_
***O. riccardii*** ** Petrulevičius et Ren, 2012**	CNU-MEC-NN2011001 P/C	0.75	Significantly basal	7 pectinate	M_1+2_, M_3_, M_4_, M_5+6_	0.8	Significantly basal	6 pectinate	M_1+2_, M_3_, M_4+5_
***O. longicauda*** ** Willmann et Novokshonov, 1998**	PIN RAN 2997/2801	N. A.	N. A.	5 pectinate	N. A.	N. A.	N. A.	N. A.	N. A.

“ca.” denotes “approximately”.

“N. A.” denotes “data not available”.

*Miriholcorpa forcipata* Wang, Shih & Ren sp. nov. (Fig. 1)

urn:lsid:zoobank.org:act:88303779-36E0-4FAC-A35A-19CFFCD231C4

#### Etymology

The specific name, *forcipata*, meaning pincer-like, referring to the fact that the male genitalia of this holotype has well-preserved extended pincer-like gonostyli.

#### Type material

Holotype CNU-MEC-NN-2012001, an almost completely preserved specimen with well-preserved genitalia and forewings, but hind wings partially preserved. Deposited at the Key Lab of Insect Evolution & Environmental Changes, College of Life Sciences, Capital Normal University (CNU), Beijing, China.

#### Age and locality

Jiulongshan Formation, late Middle Jurassic (the Callovian–Bathonian boundary), Daohugou Village, Shantou Township, Ningcheng County, Inner Mongolia, China.

#### Diagnosis

As for the genus by monotypy.

#### Description

Lateral view (Figs. 1A–C). Length ca. 32.7 mm (from head to genital end), male. Left forewing, abdomen segments and genitalia well preserved. Right forewing and right hind wing overlapped. Venation of both hind wings partially preserved.

Head. Vertex of head raised. Antenna filiform, only base preserved, scape short, pedicel and several flagellums visible. Compound eyes big and oval.

Thorax. Apparently same as generalized panorpoid morphology. Prothorax, mesothorax and metathorax discernible.

Forewing. Forewing broad, with scattered light spots and fascia, outer margin indistinct, anterior edge slightly convex, apical margin rounded, length ca. 16.6 mm, width ca. 5.5 mm (Figs. 1A–C, D). Sc extremely long, almost reaching midsection of pterostigma. Three costal crossveins present, including humeral vein. R close to Sc in basal part, forking at a quarter of wing. Terminal of R_1_ curving around pterostigma, meeting C on anterior wing margin well before apex. Several crossveins present between Sc and R_1_, number indeterminate. Rs_1+2_ with five pectinate branches ending at apical wing margin. Rs_3+4_ with two branches. Stem of Rs_1+2_ 1.65 times as long as stem of Rs_3+4_. One crossvein between Rs_1+2_ stem and R_1_, present after Rs furcation. M and R relatively parallel and close in stem base. M furcation at the same level as Rs. M_1_ simple, M_2+3_ and M_4+5_ originated from a stem. M_2+3_ stem slightly shorter than half of M_1_, M_4+5_ stem longer than one third of M_2+3_ stem. One crossvein present between M_1_ and M_2+3_ stem, before M_2+3_ furcation. Cu_1_ curved basally, then straight, terminating on margin mid-wing. Cu_2_ close to Cu_1_ at base, then parallel with Cu_1_ to margin just before mid-wing. Only one crossvein present between Cu_1_ and M, crossvein cu_1_-cu_2_ also present. 1A mostly straight with a slight bend at base. 2A parallel with 1A. 3A preserved clearly, bending posteriorly at base and reaching wing margin at one tenth of wing length. Two crossveins between 1A and 2A, only one crossvein between 2A and 3A.

Hind wing. Left hind wing partially preserved, right hind wing covered by the right forewing, incomplete (Figs. 1A–C, E). Length ca. 15.2 mm, anterior edge slightly sharp, with only portion of R and M. Stem of Rs_1+2_ 1.36 times as long as stem of Rs_3+4_. Rs furcation slightly basal to M furcation. M_1_ and M_2_ originated from one stem. M_1+2_ furcation distal to Rs_3+4_ branching. M_3_ simple, but M_4_ partially preserved. Remaining part of M indiscernible.

Abdomen. A6 3.7 mm long, spurs at the terminal part of A6 absent (Fig. 1F), A7 4.7 mm long. A8 6.9 mm long, ca. 1.5 times as long as that of A7, slightly expanded cone-like at distal end.

Genitalia. Genital bulb distinctively enlarged (Figs. 1G, H). Epandrium and hypandrium at dorsal and ventral sides, with gonopods positioned in between. Basal gonocoxite and distal gonostyli preserved. Gonostyli partially overlapped and extended pincer-like, length ca. 2.8 mm, slender, with tufts of dense brunet setae on their surface.

Mecoptera Packard, 1886

Family Incertae sedis.

*Fortiholcorpa* Wang, Shih & Ren gen. nov.

urn:lsid:zoobank.org:act:A9AA8007-451F-44B2-9CB9-FD7C732F4288

#### Type species

*Fortiholcorpa paradoxa* Wang, Shih & Ren sp. nov.

#### Etymology

The generic name is a compound word derived from a Latin word, *Fort*- meaning strong, referring to the exceedingly elongate terminal abdominal segments and enlarged genitalia, and *Holcorpa*, the name of type genus of Holcorpidae.

#### Diagnosis

Both fore- and hind wings have 5-branched M veins. Forewing Cu_1_ terminating on margin distinctly beyond mid-wing. Hind wing Rs_1+2_ furcation at same level of Rs_3+4_ furcation. Terminal abdominal segments (A6 to A8) elongate exaggeratedly, A8 slightly longer than A7, both exceedingly elongate, much longer than A6. Male genital bulb enlarged and gonostyli extended.

#### Remarks

*Fortiholcorpa* gen. nov. has affinities with Holcorpidae: (1) both fore- and hind wings have 5-branched M veins; (2) terminal abdominal segments (A6 to A8) elongate exaggeratedly; and (3) genital bulb enlarged and gonostyli extended. But, the new genus is different from the type genus *Holcorpa* of Holcorpidae in branching pattern of hind wing M veins, length of A8 slightly longer than that of A7 (vs. A8 distinctively longest); and lacking spurs at the terminal part of A6 (vs. two spurs present). In addition, it is not discernible whether dististyli basal tooth is lacking [5], [7]. Therefore, we deem *F. paradoxa* gen. et sp. nov. as Family Incertae sedis.

This new genus is distinguished from *Holcorpa* by the following characters: Forewing: long and narrow [vs. broader and much shorter]; Rs_3+4_ furcation slightly after the mid-wing [vs. Rs_3+4_ furcation ca. three quarters of wing length from wing base]; Cu_1_ terminating on posterior margin distinctly beyond mid-wing, slightly bending at base [vs. Cu_1_ terminating on margin of mid-wing, straight at the apical portion]. Hind wing: the stem of Rs_1+2_ as long as the stem of Rs_3+4_ [vs. the stem of Rs_1+2_ nearly half as long as the stem of Rs_3+4_]; M_3+4_ forking to M_3_ and M_4_, M_5_ simple [vs. M_3_ simple, M_4+5_ forking to M_4_ and M_5_] (Table 1). Abdomen: A8 slightly longer than that of A7, exceedingly elongate, ca. 3 times as long as A6 [vs. A6 and A7 nearly same in length, A8 ca. 1.5 times as long as that of A6 and A7] [5], [7].

The new genus is distinguished from *Miriholcorpa* Wang, Shih & Ren gen. nov. by the following characters: Forewing: long and narrow [vs. broader and much shorter]; Cu_1_ terminating at posterior margin distinctly beyond mid-wing [vs. Cu_1_ terminating at margin of mid-wing]. Hind wing: long, apical margin relatively round [vs. much shorter, apical sharp]; R furcation at ca. a quarter of the wing near the base [vs. R furcation considerably close to the base]; the stem of Rs_1+2_ as long as the stem of Rs_3+4_ [vs. the stem of Rs_1+2_ nearly 1.36 times as long as the length of Rs_3+4_ stem] (Table 1). Abdomen: A7 and A8 nearly the same in length, exceedingly elongate, ca. 3 times as long as A6 [vs. A6 slightly shorter than A7, A8 ca. 1.5 times as long as that of A7].

*Fortiholcorpa paradoxa* Wang, Shih & Ren sp. nov. (Fig. 2)

urn:lsid:zoobank.org:act:6F094D36-BEF9-4D07-91BE-0EF7E7093C34

#### Etymology

The specific name, *paradoxa*, means fantastic, referring to the rare and fantastic terminal segments of abdomen which are extremely extended.

#### Type material

Holotype, CNU-MEC-NN-2012002 P/C, a male specimen with four wings overlapping, and terminal abdominal segments exaggeratedly extended. Deposited at the Key Lab of Insect Evolution & Environmental Changes, College of Life Sciences, Capital Normal University (CNU), Beijing, China.

#### Age and locality

Jiulongshan Formation, late Middle Jurassic (the Callovian–Bathonian boundary), Daohugou Village, Shantou Township, Ningcheng County, Inner Mongolia, China.

#### Diagnosis

As for the genus by monotypy.

#### Description

A male holotype preserved in lateral view (Figs. 2A–C), length ca. 73.5 mm (from head to genital end), four wings overlapping. Most of abdomen preserved under wings except for terminal segments. A7 and A8 exceedingly elongate.

Head. Antenna filiform, with a short and stout scape, terminal part not discernible. Compound eyes big and oval.

Thorax. Poorly preserved, only mesothorax and metathorax recognizable. Femora, tibiae and tarsi of fore legs partially discernible. Basal part of femora of middle leg distinguishable. Femora of hind legs ca. 3.9 mm.

Forewing. Forewing partly preserved on top of overlapping four wings (Figs. 2A, C, D), length ca. 20.5 mm, width unknown. Part of Rs_3+4_ visible. M with five branches, bifurcations untraceable, One crossvein present between M_3_ and M_4_, M_4_ and M_5_, and M_5_ and Cu_1_ respectively. Base of Cu_1_ and Cu_2_ not preserved. Cu_1_ with a distinct bend at three quarters of Cu_1_ length from the wing base, terminating at posterior margin far beyond mid-wing. Part of Cu_2_ preserved mostly straight and terminating at middle of wing margin. 1A and 2A mostly straight, 2A parallel with 1A. 3A bending posteriorly at base and reaching wing margin at one sixth of wing length. Two crossveins between 2A and 3A.

Hind wing. Most veins discernible (Figs. 2A, C, E), length ca. 20.1 mm, slightly shorter than forewing (20.5 mm). C invisible. Sc furcation slightly after mid-wing, branching into Sc_1_ and Sc_2_. R_1_ single. Rs_1+2_ with five pectinate branches, Rs_3+4_ with two branches, Rs_1+2_ furcation at the same level as Rs_3+4_ furcation. M with five branches. M_1+2_ and M_3+4_ originated from a stem, M_1+2_ stem three times as long as M_3+4_ stem. M_5_ single, while origination and basal part of M_5_ not discernible, possibly joining M as the first branching where M appearing to bend slightly before forking into M_1+2_ and M_3+4_. Cu_1_ mostly straight with a bend at three quarters of Cu_1_ length from the wing base. Cu_2_ with only distal end preserved, terminating at margin slightly before mid-wing. Origins of R, Cu_1_ and 1A quite close at wing base. 1A and 2A straight, relatively parallel from base to distal end. 3A preserved clearly, bending posteriorly at base and reaching wing margin at one sixth of wing length. One crossvein present between 2A and 3A near distal end of 3A.

Abdomen. A6 7.0 mm long, tergal spurs on A6 absent (Fig. 2F). A7 and A8 exceedingly elongate and slender. A7 21.1 mm long, slightly curved at base, expanded cone-like at distal end; tergite and sternite of A7 fused into a seam line (Figs. 2A, C and F). A8 21.8 mm long, slightly curved at the middle section.

Genitalia. Preserved in ventral view, genital bulb large. Hypandrium with a pair of hypovalvae discernible, gonocoxite and gonostyli visible (Figs. 2G, H). Gonostyli elongated pincer-like, length ca. 3.8 mm, broad at base and tapering towards apex, distal part curving inward.

#### Remarks

The fusion line of tergite and sternite in A7 has been first reported for *O. longicauda* Willmann et Novokshonov, 1998 [26]. Willmann and Novokshonov found this interesting that this line of fusion is gone in the Panorpidae, although still apparent in the Panorpodidae. Archibald stated that “In the male specimen of *H. maculata*, preservation precludes determination of the existence of such a seam, however, in the holotype of *H. dillhoffi*, this seam is possibly preserved, although, if so, faintly, not with enough clarity for certainty” [7]. For *F. paradoxa* gen. et sp. nov., this seam line is distinctly preserved on the fossil and clearly shown (Figs. 2A, C and F).

## Discussion

Compared the two new genera with the type genus *Holcorpa* of Holcorpidae, there are several different characters: the shape of wings; the branching patterns of forewing M veins for *Miriholcorpa* gen. nov. and hind wing M veins for *Fortiholcorpa* gen. nov.; spurs absent at the end of A6 (sixth abdominal segment); and the relative length ratio of A6, A7 and A8. In addition, it is not discernible whether they don’t have dististyli basal tooth [5], [7]. Furthermore, it is uncertain whether hind wing of *Miriholcorpa* gen. nov. has 5-branched M veins. Therefore, we deem these two new genera as Family Incertae sedis, pending future discovery of new fossil specimens with clear preservation of wing venation.

The holcorpid fossils have a unique combination of two characters which can easily separate holcorpids from other mecopterans. The first is a plesiomorphy that vein M with five branches in both fore- and hind wings, which is present in only two other family, Choristopsychidae Martynov, 1937 [30] and Dinopanorpidae Archibald, 2005 [31]. The second is the A6 to A8 exceedingly elongate and male genitalia enlarged, which are also present in some species of Orthophlebiidae from the late Middle Jurassic of Inner Mongolia, China (eg. *Orthophlebia nervulosa* Qiao, Shih et Ren, 2012 and *O. riccardii* Petrulevičius et Ren, 2012) [32], [33] and from the Upper Jurassic of Karatau (Kazachstan) (eg. *Orthophlebia longicauda* Willmann et Novokshonov, 1998 [26]) and some species of extant *Leptopanorpa* MacLachlan, 1875 of Panorpidae, (eg. *L. filicauda* Lieftinck, 1936, *L*. *longicauda* Weele, 1909, *L. nematogaster* MacLachlan, 1869, and *L. robusta* Lieftinck, 1936) [34]. Since the degree of elongation and relative lengths of A6, A7 and A8 are different for species in these three families and the two new genera, we summarized comparison data for males in Table 2. We listed the total lengths of A7–A8, A6–A8, A1–A6 and A1–A5 respectively, and calculated length ratio data of A7–A8/A1–A6 and A6–A8/A1–A5.

**Table 2:** Comparison of fossil and extant species with extremely lengthened male abdominal segments A6 to A8.

		Material ID number	Length of A6 (mm)	Length of A7 (mm)	Length of A8 (mm)	Total length of A7–A8 (mm)	Total length of A1–A6 (mm)	Length Ratio of A7–A8/A1–A6	Total length of A6–A8 (mm)	Total length of A1–A5 (mm)	Length Ratio of A6–A8/A1–A5
***Holcorpa*** ** Scudder, 1878, Holcorpidae Willmann, 1989**											
***H. maculosa*** ** Scudder, 1878**	AMNH 18887 (part) and UCM4494 (counterpart)		10.0	10.0	16.3	26.3	19.5	1.4	36.3	9.5	3.8
***H. dillhoffi*** ** Archibald, 2010**	RBCM-EH-2008-018-0001 (part only)^1^ *		ca. 10.4	8.4	13.4	21.8	ca. 22.2	ca. 1.0	ca. 32.2	ca. 11.8	ca. 2.7
***Fortiholcorpa*** ** gen. nov., Family Incertae sedis**											
***F. paradoxa*** ** sp. nov.**		CNN-MEC-NN-2012002 p/c	7.0	21.1	21.8	42.9	17.7	2.4	49.9	10.7	4.7
***Miriholcorpa*** ** gen. nov., Family Incertae sedis**											
***M. forcipata*** ** sp. nov.**		CNN-MEC-NN-2012001 (part only)	3.7	4.7	6.9	11.6	11.3	1.0	15.3	7.6	2.0
***Orthophlebia*** ** Westwood, 1845, Orthophlebiidae Handlirsch, 1906**											
***O. nervulosa*** ** Qiao, Shih et Ren, 2012**		CNU-MEC-NN-2009617 (part only)	3.9	3.7	2.8	6.5	12.7	0.5	10.4	8.8	1.2
***O. riccardii*** ** Petrulevičius et Ren, 2012**		CNU-MEC-NN2011001 p/c	11.0	6.6	3.0	9.6	21.9	0.4	20.6	10.9	1.9
***O. longicauda*** ** Willmann et Novokshonov, 1998**		PIN RAN 2997/2801^2^ *	ca. 5.3	5.6	8.1	13.7	14.7	0.9	ca. 19.0	ca. 9.4	ca. 2.0
***Leptopanorpa*** ** MacLachlan, 1875, Panorpidae Stephens, 1836**											
***L. filicauda*** ** Lieftinck, 1936**		EXTANT^3^ *	3.8^12^ *	6.7	6.2	12.9	9.0	1.4	16.7	5.2	3.2
		EXTANT^4^ *	4.8^12^ *	8.5	7.8	16.3	9.0	1.8	21.1	4.2	5.0
		EXTANT^5^ *	5.4^12^ *	9.5	9.3	18.8	11.0	1.7	24.2	5.6	4.3
***L. longicauda*** ** Weele, 1909**		EXTANT^6^ *	2.3^12^ *	4.3	4.0	8.3	8.0	1.0	10.6	5.7	1.9
		EXTANT^7^ *	4.3^12^ *	8.2	7.1	15.3	11.0	1.4	19.6	6.7	2.9
		EXTANT^8^ *	2.9^12^ *	5.5	4.5	10.0	9.5	1.1	12.9	6.6	2.0
***L. nematogaster*** ** MacLachlan, 1869**		EXTANT^9^ *	2.4^12^ *	4.3	4.1	8.4	7.5	1.1	10.8	5.1	2.1
		EXTANT^10^ *	3.2^12^ *	5.8	5.5	11.3	10.2	1.1	14.5	7.0	2.1
***L. robusta*** ** Lieftinck, 1936**		EXTANT^11^ *	2.4^12^ *	4.6	4.6	9.2	8.9	1.0	11.6	6.5	1.8

1 *The abdomen of the specimen is disarticulated between the 5^th^ and 6^ th^ segments. For length of A1 to A5, it is measured to the ending of the 5^th^ segment. “ca.” denotes “approximately”.

2 *The 5^th^ and 6^ th^ abdominal segments are not clearly marked in Fig. 23 of [26] It is assumed that the 6^ th^ segment starts at the point of sudden narrowing of abdomen, “ca.” denotes “approximately”.

3 *W. Java, eastern slope of Mt. Gedeh, Tjibeureum (ncar Tjibodas), 1700 m, June 1932, L. J. TOXOPEUS,

4 *Tjibodas, 1700 m, no. 24, 1923, KARNY.

5 *E. JACOBSON, Goenoeng Gedeh, Java, March 1911 (printed) *Leptopanorpa longicauda* WEELE (PETERSEN'S handwriting) “Fig. Catal SELYS” (printed), in coll. ESBEN PETERSEN.

6 *W. Java, northwestern slope of Mt. Gedeh, 800 m, Tapos, 1932–1934, all the year round, L. G. E. KALSHOVEN and native coll., smallest specimen.

7 *W. Java, northwestern slope of Mt. Gedeh, 800 m, Tapos, 1932–1934, all the year round, L. G. E. KALSHOVEN and native coll., largest specimen.

8 *Djampang Koelon, Mt. Malang, 600 m, May 1934, M. E. WALSH.

9 *W. Java, eastern slope of Mt. Gedeh, Tjibodas, 14–1500 m, Aug. 1921, 1923, H. H. KARNY, July 28 and Dec. 25, 1930, Aug. 9, 1931, and Jan. 2, 1936, M. A. LIEFTINCK, Sept. 8, 1931, T. VAN BENTHEM JUTTING, June 26, Dec. 28, 1933, L. J. TOXOPEUS, and May 22–23, 1935, J. VAN DER VECHT; W. Java, western slope of Mt. Goentoer, Kamodjang, 1450 m, April 21, 1930, M. A. LIEFTINCK, and May 1935, H.OVERBECK; smallest specimen.

10 *W. Java, eastern slope of Mt. Gedeh, Tjibodas, 14–1500 m, Aug. 1921, 1923, H. H. KARNY, July 28 and Dec. 25, 1930, Aug. 9, 1931, and Jan. 2, 1936 M. A. LIEFTINCK, Sept. 8, 1931, T. VAN BENTHEM JUTTING, June 26, Dec. 28, 1933, L. J. TOXOPEUS, and May 22–23, 1935, J. VAN DER VECHT; W. Java, southern slope of Mt. Tangkoeban Prahoe, 1500 m, Sept. 10, 1929, Dec. 7, 1933, Jan. 4, 1934, F. C. DRESCHER; largest specimen.

11 *Central Java, southern slope of Mt. Slamat, Batoerraden, 950 m, Oct. 21, 1933, M. A. LIEFTINCK. The data is the average value of the reported length range of the specimens.

12 *Length of 6^th^ segment (A6) of each specimen is estimated by using the proportion between A7 and A6 as shown in Plate 7 and Plate 8 of [34].

The data in Table 2 show that three representative species of orthophlebiids, with A6 longer than A7 or A8 for two species and A8 longer than A6 or A7 for one, have the least degree of elongation for A7 and A8 and the lowest length ratios of A7–A8/A1–A6, 0.4 to 0.9. and A6–A8/A1–A5 range from 1.2 to ca. 2.0. For four species of extant panorpids, the A8 is slightly shorter than A7, but both A7 and A8 are significantly longer than A6. The length ratios of A7–A8/A1–A6 range from 1.0 to 1.8, while the ratios of A6–A8/A1–A5 range from 1.8 to 5.0. Especially, two specimens of *L. filicauda* have A7 and A8 extended significantly with A7–A8/A1–A6 ratios of 1.7 and 1.8 and A6–A8/A1–A5 ratios of 4.3 and 5.0 (the highest). As for holcorpids, the A8 is longer than A6 or A7, A6 is equal to or slightly shorter than A7 in contrast to *F. paradoxa* gen. et sp. nov. with A6 ca. one third of A7 in length. For holcorpids, the length ratios of A7–A8/A1–A6 range from 1.0 to 1.4 and A6–A8/A1–A5 ratios of 2.7 to 3.8. For *F. paradoxa* gen. et sp. nov., the length ratio of A7–A8/A1–A6 is 2.4 (the highest) and A6–A8/A1–A5 ratio is 4.7. For *M. forcipata* gen. et sp. nov., the length ratio of A7–A8/A1–A6 is 1.0 and A6–A8/A1–A5 ratio is 2.0, similar to those of *H. dillhoffi* and *O. longicauda*. This set of data clearly show that *F. paradoxa* gen. et sp. nov. has the most exaggeratedly elongate abdominal segments of A7–A8 but relatively short A6.

The scorpion-like terminalia with less exaggerated elongation of A6–A8 in typical extant *Panorpa* males, Fig. 3, is known to be used for aggressive fighting between males and assisting mating and copulation with females [35], [36]. Thornhill [36] reported *Panorpa* males use three alternative mating tactics: presenting a dead arthropod to a female and allowing the female to feed in exchange for copulation is the most successful tactics, followed by presenting a salivary mass. The third and the least successful tactics is forced copulation without any nuptial food offerings. Males use large and muscular genitalia equipped with a genital bulb and a pair of sharp claspers to fight and to deter other males in the competition of available nuptial food. He also confirmed that large males have an advantage in male-male competition for arthropods by observation of interactions of males of known size around dead crickets in the enclosures [36].

## Conclusions

Most Mesozoic fossil male orthophlebiids [26], [32], [33] have similar scorpion-like terminalia in size and proportion to other body parts as those of typical extant *Panorpa* males. However, extinct holcorpids, some extant *Leptopanorpa* of panorpids and these two new genera have extremely long and extended terminal abdominal segments and enlarged male genitalia, especially in *F. paradoxa* gen. et sp. nov. with the highest ratio of A7–A8/A1–A6 at 2.4 and *L. filicauda* with highest ratio of A6–A8/A1–A5 at 5.0 as aforementioned. The sixth abdominal segment of *F. paradoxa* gen. et sp. nov. is covered by wings and the seventh and eighth abdominal segments curving upward with enlarged male organs (Figs. 2A, C) suggesting competitive and/or sexual display. The same posture has been reported for extant *L. longicauda* as resting position in Fig. 7 of [34].

We propose a likely evolutionary scenario that the elongate terminal abdominal segments of *F. paradoxa* gen. et sp. nov. and *M. forcipata* gen. et sp. nov. acted as a symbol of large body size to intimidate competitive males and/or as a display to attract potential mates starting in (or before) the late Middle Jurassic. Judging by the thin and narrow tubular structure of A7 and A8 segments preserved on the fossils, these two new genera and holcorpids found in the Early and Late Eocene might not have had the robustness and power in these segments to engage fierce fighting with other males. For these insect taxa, a separate evolutionary pathway emphasizing “form” being more important than “function” resulted in such exaggerated terminalia. Extremely long terminal abdominal segments, on the other hand, might have hindered the survivability of individuals due to more visibility to predators and less mobility to evade attacks. It is interesting to note that the long period of their existence suggests that the sexual display and sexual selection due to extremely elongated male abdominal and sexual organs outweighed the negative impact of bulky body and poor mobility in the evolutionary process. This character of extremely elongate terminal abdominal segments is also present in some species of the extant *Leptopanorpa* of Panorpidae, which might have been resulted from an independent evolutionary convergence or a genetic trait passed on from a common ancestor of Panorpidae, Holcorpidae and these two new genera.
